# Shallow Acupuncture for brainstem infarction: a randomized controlled trial protocol

**DOI:** 10.3389/fneur.2026.1849749

**Published:** 2026-07-10

**Authors:** Hongge Zuo, Yan Huang, Weikang Huang, Jiahui Lin, Jinyuan Fang, Zhenhua Xu

**Affiliations:** 1 The Second Clinical College of Guangzhou University of Chinese Medicine, Guangzhou, China; 2 Department of Acupuncture Rehabilitation, Ganzhou Hospital of Traditional Chinese Medicine, Ganzhou, China; 3 Shenzhen Traditional Chinese Medicine Hospital, Shenzhen, China

**Keywords:** brainstem infarction, diffusion tensor imaging, functional connectivity, resting-state functional magnetic resonance imaging, Shallow Acupuncture, stroke rehabilitation, study protocol

## Abstract

**Background:**

Brainstem infarction is a clinically important subtype of posterior circulation infarction and is often associated with disability and poor functional recovery. Disease-specific therapeutic strategies remain limited, and rehabilitation-oriented evidence specifically targeting functional recovery in this population remains insufficient. Acupuncture has been studied and applied as an adjunctive approach for stroke rehabilitation in multiple regions; however, high-quality evidence for its use in brainstem infarction remains scarce. This trial primarily aims to evaluate whether adjunctive Shallow Acupuncture improves activities of daily living in patients with brainstem infarction; as an exploratory objective, it will examine associated neural changes assessed by resting-state functional magnetic resonance imaging (rs-fMRI) and diffusion tensor imaging (DTI).

**Methods and analysis:**

This is a single-center, randomized, assessor- and statistician-blinded, pragmatic add-on controlled trial. A total of 62 patients with brainstem infarction will be randomly assigned in a 1:1 ratio to a conventional acupuncture group or an adjunctive Shallow Acupuncture group. In keeping with routine inpatient practice in the acupuncture department, both groups will receive standard medical management, routine rehabilitation, and conventional acupuncture, while the adjunctive Shallow Acupuncture group will additionally receive Shallow Acupuncture once daily for 14 consecutive days. The primary clinical outcome will be activities of daily living, assessed by the Barthel Index. Secondary clinical outcomes will include the National Institutes of Health Stroke Scale and the modified Rankin Scale. Exploratory indicators will include measures derived from rs-fMRI and DTI. rs-fMRI analyses will focus primarily on resting-state functional connectivity, whereas DTI analyses will focus primarily on fractional anisotropy; mean diffusivity will be examined as an exploratory diffusion metric. Clinical outcomes will be assessed at baseline, post-treatment, and 1-month follow-up, and neuroimaging assessments will be performed at baseline and post-treatment.

**Ethics and dissemination:**

This study has been approved by the Ethics Committee of Guangdong Provincial Hospital of Chinese Medicine (approval numbers: YE2024-202-01 and YE2024-203-01). The findings will be disseminated through peer-reviewed publications and academic conferences.

**Clinical trial registration:**

https://itmctr.ccebtcm.org.cn/zh/index.html. Identifier, ITMCTR2024000456.

## Introduction

Brainstem infarction, a subtype of ischemic stroke, refers to ischemic necrosis of the midbrain, pons, or medulla oblongata caused by stenosis or occlusion of the vertebrobasilar artery and its branches, accounting for approximately 10–15% of all ischemic stroke (1). Located at the base of the brain, the brainstem serves as a major pathway for motor and sensory tracts, harbors nuclei and fibers of ten cranial nerves, and regulates core functions such as cardiac and respiratory activity, consciousness, and sleep–wake cycles (2). Because of its structural complexity and critical physiological roles, even small lesions in this region can provoke severe and heterogeneous clinical manifestations (3). Compared with ischemic strokes in other locations, brainstem infarction is characterized by a more abrupt onset and a more severe clinical course (4) and represents a significant contributor to global stroke-related morbidity and mortality (1). However, despite its clinical importance and generally poor prognosis, research specifically focusing on brainstem infarction remains limited, and disease-specific therapeutic strategies are still lacking (5).

Current management of brainstem infarction largely follows general treatment principles for ischemic stroke, including acute reperfusion therapy, neurosurgical procedures, and pharmacological treatment (5). Although acute reperfusion therapies remain important, their applicability is constrained by narrow therapeutic time windows, restricted indications, and uncertain benefit in some patients (6–8). Neurosurgical intervention is generally reserved for selected life-threatening conditions rather than routine management (5), whereas pharmacological treatment mainly focuses on secondary prevention and provides limited direct benefit for functional recovery. Despite standard acute and medical management, many patients with brainstem infarction continue to experience disabling deficits and require rehabilitation-oriented management.

Current stroke rehabilitation strategies are typically individualized according to functional deficits and rehabilitation needs rather than determined solely by infarct location (9). As a result, patients with brainstem infarction are often managed within broader stroke rehabilitation frameworks. However, lesions involving the midbrain, pons, or medulla may result in heterogeneous and disabling functional deficits, including dysphagia, dysarthria, vertigo or dizziness, impaired balance, ataxia, limb weakness, and other related dysfunctions (1). In addition, posterior circulation infarction, including brainstem infarction, remains relatively under-researched compared with anterior circulation stroke (5). Therefore, high-quality rehabilitation-oriented evidence specifically targeting functional recovery after brainstem infarction remains limited. Accordingly, there remains a clear need to explore adjunctive strategies that may promote functional recovery after brainstem infarction.

Acupuncture has been investigated as an adjunctive approach for stroke rehabilitation in studies from multiple regions (10). In China, acupuncture is also recommended in the Chinese Guidelines for the Diagnosis and Treatment of Acute Ischemic Stroke 2023 as an adjunctive therapy for acute ischemic stroke (Class IIb recommendation, Level B evidence) (11). In routine clinical practice in Chinese tertiary hospitals of traditional Chinese medicine, conventional acupuncture is commonly incorporated into integrative rehabilitation for stroke. Although previous studies suggest that acupuncture may improve neurological symptoms after cerebral infarction, high-quality evidence specifically targeting posterior circulation infarction, particularly brainstem infarction, remains scarce (12–14).

In this study, we employ Shallow Acupuncture, a superficial needling technique in which the needle is inserted at a small angle and limited depth into the subcutaneous tissue or superficial muscle layer (15). Unlike conventional acupoint-based acupuncture, this technique emphasizes the identification and stimulation of pathological response points (16). These points are defined here as clinically identifiable reactive areas on the body surface that may reflect altered sensitivity, tenderness, or local neurovascular reactivity. For patients with brainstem infarction, the pathological basis is closely related to ischemia within the vertebrobasilar arterial territory (1). Previous studies have suggested that acupuncture applied to cervico-occipital regions may be associated with changes in vertebrobasilar blood flow and functional connectivity (17, 18), providing a regional neurovascular and neuroimaging rationale for considering cervico-occipital region stimulation. In our preliminary clinical observations, pathological response points in patients with brainstem infarction frequently clustered in the temporal and occipital regions, and stimulation of these regions appeared to be associated with improvement in symptoms such as dizziness. This observation further informed the selection of the temporal and occipital regions in the present protocol. In addition, our previous randomized clinical study reported that Shallow Acupuncture improved dizziness-related outcomes in patients with posterior circulation ischemic vertigo, providing clinical evidence for the application of this technique in posterior circulation ischemia-related symptoms (19). Taken together, these observations provide a rationale for the design of a disease-oriented and lesion-directed Shallow Acupuncture protocol for brainstem infarction. However, because the current supporting evidence remains indirect, the present protocol is best regarded as hypothesis-generating and requires prospective evaluation. Rigorous randomized controlled evidence remains lacking regarding whether adjunctive Shallow Acupuncture confers additional clinical benefit in brainstem infarction and whether any such benefit is accompanied by measurable neural changes.

To investigate whether adjunctive Shallow Acupuncture is accompanied by treatment-related neural changes after brainstem infarction, rs-fMRI and DTI provide complementary approaches for evaluating brain network reorganization and white-matter microstructural changes. Blood oxygen level–dependent functional magnetic resonance imaging (BOLD-fMRI), which indirectly reflects neuronal activity by detecting blood oxygen–dependent signals, has become a key tool for investigating brain functional activity (20). Previous studies have shown that patients with brainstem infarction exhibit abnormalities in resting-state network connectivity, and that these changes may be associated with functional recovery (21–23). Diffusion tensor imaging (DTI), by quantifying the diffusion properties of water molecules, enables assessment of white matter tract integrity (24). Fractional anisotropy (FA), a commonly used DTI parameter, has been shown to decrease after brainstem infarction, particularly in the corticospinal tract (CST), and subsequent FA recovery may be associated with more favorable motor outcomes (25–27).

Together, rs-fMRI and DTI may help characterize brain network reorganization and white-matter microstructural changes after brainstem infarction, thereby providing a multimodal framework for exploring clinical–imaging associations in response to adjunctive intervention. Against this background, the present randomized controlled trial is designed to evaluate the adjunctive effectiveness of Shallow Acupuncture in patients with brainstem infarction within routine integrative inpatient care. We hypothesize that, compared with standard care plus conventional acupuncture alone, adjunctive Shallow Acupuncture will be associated with greater improvement in activities of daily living and may be accompanied by changes in resting-state functional connectivity and white-matter microstructural integrity as assessed by rs-fMRI and DTI.

## Methods and analysis

### Study design

**Figure 1:**
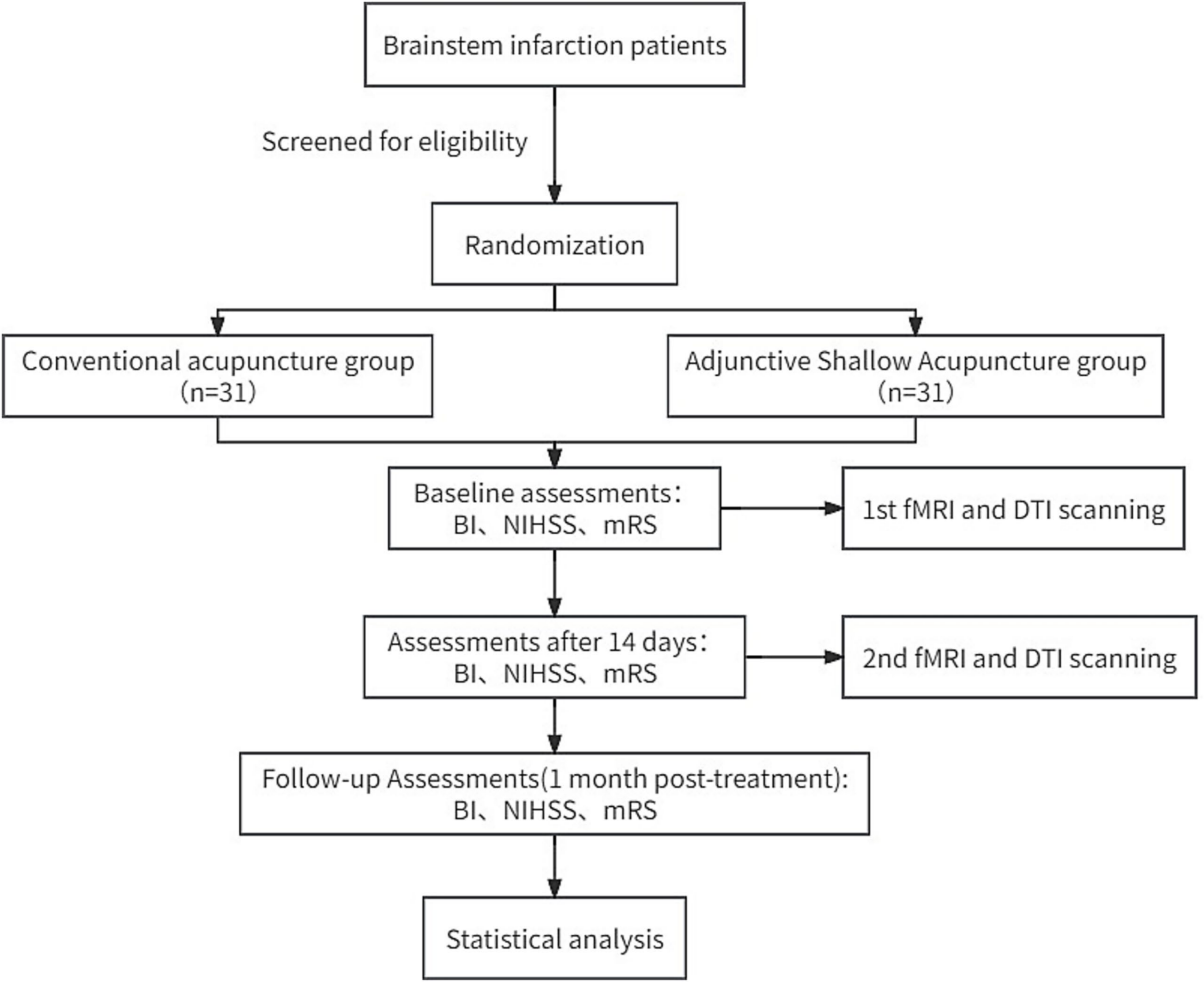
Flowchart of the randomized controlled trial. BI, Barthel Index; NIHSS, National Institutes of Health Stroke Scale; mRS, modified Rankin Scale; fMRI, functional magnetic resonance imaging; DTI, diffusion tensor imaging.

This is a single-center, randomized, assessor- and statistician-blinded, pragmatic add-on trial, in which a predefined subgroup of participants will undergo rs-fMRI and DTI assessment. All study procedures adhere to the Standard Protocol Items: Recommendations for Interventional Trials (SPIRIT) guidelines. Figure 1 outlines the study flow, and Table 1 details the schedule of enrolment, interventions, and assessments.

**Table 1:** Schedule of enrolment, interventions, and assessments.

	Study period				
	Screening/Enrolment	Baseline (Day 0)	Treatment period (Day 1–14)	Post-treatment (Day 14)	Follow-up (1 month post-treatment)
Eligibility assessment	**×**				
Informed consent	**×**				
Brain MRI confirmation of brainstem infarction	**×**				
Demographic and clinical characteristics		**×**			
Documentation of pathological response points		**×**	**×**		
Randomization		**×**			
Standard medical management			**×**		
Routine rehabilitation			**×**		
Conventional acupuncture (both groups)			**×**		
Shallow Acupuncture (adjunctive Shallow Acupuncture group)			**×**		
BI		**×**		**×**	**×**
NIHSS		**×**		**×**	**×**
mRS		**×**		**×**	**×**
fMRI and DTI (imaging subgroup only)		**×**		**×**	
Adverse event/safety monitoring			**×**	**×**	

BI, Barthel Index; NIHSS, National Institutes of Health Stroke Scale; mRS, modified Rankin Scale; fMRI, functional magnetic resonance imaging; DTI, diffusion tensor imaging.

The study protocol has been reviewed and approved by the Ethics Committee of Guangdong Provincial Hospital of Chinese Medicine (Ethics Reference No: YE2024-202-01, YE2024-203-01). The trial has also been registered in the International Traditional Chinese Medicine Clinical Trial Registry (ITMCTR; registration number: ITMCTR2024000456).

### Setting and recruitment

This trial will be conducted in the inpatient Acupuncture Department of The Second Affiliated Hospital of Guangzhou University of Chinese Medicine (Guangzhou, China) from August 2025 to November 2026. During this period, 62 patients with brainstem infarction and 20 healthy volunteers will be recruited. The healthy volunteers will serve as a neuroimaging comparison group for the assessment of brain network and white-matter changes associated with brainstem infarction. Healthy volunteers will not undergo randomization or intervention and will receive a single rs-fMRI and DTI assessment for neuroimaging comparison. Because the study recruits inpatients from the acupuncture department, conventional acupuncture is incorporated into routine integrative stroke care in this clinical setting. Accordingly, the present trial is designed to evaluate the adjunctive effectiveness of Shallow Acupuncture on top of standard care and conventional acupuncture under real-world inpatient conditions.

### Inclusion criteria

Patients meeting all of the following criteria will be included:

1. Diagnosis of ischemic stroke consistent with the Guidelines for Primary Care of Ischemic Stroke (2021) (28), and brain MRI confirming a single ischemic infarction involving the brainstem, with the lesion confined to one side and not crossing the midline.
2. Men or women aged 50–80 years, with a first-ever stroke and a disease duration ≤ 6 months.
3. NIHSS score ≥ 2, and the presence of at least one assessable brainstem-related neurological deficit, such as dysphagia, dysarthria, vertigo, balance impairment, or ataxia.
4. Clinically stable, without disturbance of consciousness, and able to understand and cooperate with study procedures.
5. Able and willing to provide written informed consent.
6. Not currently participating in any other clinical study.
7. Right-handed prior to stroke onset, as assessed by the Chinese version of the Edinburgh Handedness Inventory (29).

### Exclusion criteria

Patients meeting any of the following criteria will be excluded:

1. Presence of any ischemic infarct lesions outside the brainstem on brain MRI.
2. Pregnancy; end-stage malignancy; history of brain surgery; or prior central nervous system diseases such as epilepsy, brain tumors, or other major neurological disorders.
3. Presence of psychiatric disorders; severe cardiac, pulmonary, hepatic, or renal dysfunction; or critical illness with unstable vital signs.
4. Skin breakdown, infection, or other local conditions at the proposed needling sites that preclude acupuncture, or inability to cooperate with the required assessments.
5. Presence of a cardiac pacemaker, vascular or other metallic implants, coronary artery bypass grafting, or any other condition that constitutes a contraindication to MRI.

### Withdrawal criteria

Subjects will be withdrawn from the study if any of the following occur:

1. Enrollment despite not meeting the predefined inclusion criteria (mis-enrollment).
2. Failure to receive the assigned intervention as per protocol, or failure to complete study documentation in a way that materially affects outcome assessment.
3. Poor treatment adherence or voluntary withdrawal from the study during the intervention period.
4. Initiation, addition, or modification of other treatments during the trial that may interfere with the evaluation of study outcomes.

### Sample size calculation

The sample size for the clinical component was calculated based on the primary clinical outcome, namely the between-group difference in Barthel Index (BI) improvement after treatment. According to previous literature (30), the mean increase in BI after conventional acupuncture was approximately 12.66 points. Because no prior study has specifically evaluated the effects of Shallow Acupuncture in patients with brainstem infarction, the expected effect size for the present study was informed by a preliminary pilot study conducted in patients with brainstem infarction treated in the Department of Acupuncture of The Second Affiliated Hospital of Guangzhou University of Chinese Medicine. In these patients, who received Shallow Acupuncture combined with conventional acupuncture, the mean increase in BI after 2 weeks of treatment was approximately 17.5 points, with a standard deviation of 5.22. The clinical sample size was estimated in PASS using the Two-Sample T-Tests Assuming Equal Variance procedure. For transparency, the conventional planning formula is shown below:


$$n=\frac{2{\left(Z_{1-\alpha /2}+Z_{1-\beta }\right)}^{2}\sigma^{2}}{\delta^{2}}$$

where n denotes the required sample size per group, *δ* denotes the expected between-group difference in BI improvement, and *σ* denotes the assumed common standard deviation. In the present study, the expected between-group difference was set at 4.84 points (17.50–12.66). Because no published estimate of the standard deviation of BI change was available for the control condition, a common standard deviation of 5.22, derived from preliminary pilot observations, was assumed for planning purposes. Using a two-sided significance level of 0.05, 90% power, and a 1:1 allocation ratio, PASS estimated that 26 participants per group (52 participants in total) would be required. Assuming a dropout rate of 15%, the final planned sample size was set at 62 patients, with 31 participants in each group.

For the neuroimaging component, a formal sample size calculation was not considered feasible because of the heterogeneity of rs-fMRI/DTI analysis methods and the exploratory nature of the imaging analyses. Therefore, the imaging sample size was determined pragmatically with reference to prior neuroimaging literature (31–33), in which 12 participants per group have often been regarded as a minimum for obtaining stable estimates. To enhance reliability and to allow for potential data loss due to motion artifacts or incomplete scans, 20 participants per group will be included in the rs-fMRI/DTI analyses.

### Randomization

A computer-generated randomization sequence will be created by an independent researcher who is not involved in participant recruitment, treatment delivery, outcome assessment, or statistical analysis. Participants will be randomly assigned in a 1:1 ratio to the conventional acupuncture group or the adjunctive Shallow Acupuncture group, with 31 patients in each group. The allocation sequence will be concealed in sequentially numbered, opaque, sealed envelopes. The envelopes will be kept by designated study personnel and opened in numerical order only after an eligible participant has been enrolled. Participants will then be assigned to the corresponding group according to the allocation indicated inside the envelope.

### Blinding

Because of the nature of the intervention, participants and acupuncturists cannot be blinded in this study. To minimize bias, several measures will be implemented. First, acupuncturists will be responsible only for treatment delivery and will not participate in participant recruitment, group allocation, outcome assessment, or data analysis. Second, all clinical outcome assessments will be performed by independent investigators who are blinded to group allocation. Third, statisticians responsible for data management and statistical analysis will remain blinded to group labels throughout the analysis process. For the neuroimaging component, imaging data processing and analysis will also be conducted by investigators who are blinded to group allocation whenever feasible.

### Interventions

All interventions will be performed by licensed acupuncturists who have completed standardized training for this trial and have at least 3 years of clinical experience. Participants in both groups will receive treatment once daily, for 2 consecutive weeks, for a total of 14 treatment sessions. In keeping with routine inpatient practice in the acupuncture department, both groups will receive standard medical management, routine rehabilitation, and conventional acupuncture. The between-group difference will be the addition of Shallow Acupuncture in the experimental arm. The overall contents of the two intervention arms are summarized in Table 2.

**Table 2:** Summary of interventions in the conventional acupuncture group and the Adjunctive Shallow Acupuncture group.

Intervention component	Conventional acupuncture group	Adjunctive Shallow Acupuncture group	Key specification
Standard medical management	Yes	Yes	Guideline-based management of ischemic stroke and vascular risk factors according to the same institutional stroke care pathway
Rehabilitation	Yes	Yes	Standard stroke rehabilitation according to the same institutional protocol
Conventional acupuncture	Yes	Yes	Once daily for 14 consecutive days; fixed prescription described in the Methods section
Shallow Acupuncture	No	Yes	Once daily for 14 consecutive days; performed at pathological response points in predefined temporal and occipital regions

### Conventional acupuncture group

Participants in the conventional acupuncture group will receive standard medical management, nursing care, routine rehabilitation, and conventional acupuncture.

For brainstem infarction and relevant comorbidities (e.g., hypertension, diabetes), both groups will receive standard medical management and nursing care in accordance with the Guidelines for Primary Care of Ischemic Stroke (2021) (28) and the Chinese Guidelines for Secondary Prevention of Ischemic Stroke and Transient Ischemic Attack 2022 (34), and according to the same institutional stroke care pathway. Standard care includes antiplatelet therapy, circulation-improving agents, maintenance of electrolyte balance, blood pressure and glucose control, and routine rehabilitation. In addition to standard stroke-related medications, symptomatic treatments and other clinically indicated medications will be prescribed according to the same institutional practice in both groups and recorded throughout the trial; for example, medications for dizziness or vertigo, which are common symptoms of brainstem infarction, will be used when clinically indicated. In both groups, routine rehabilitation will be delivered by rehabilitation therapists according to the same institutional rehabilitation protocol and individualized according to each patient’s neurological deficits. The rehabilitation program may include limb motor training, balance and gait training, activities of daily living training, swallowing training, speech- or dysarthria-related training, and other task-oriented functional exercises when clinically indicated. Throughout the study period, all concomitant medications and rehabilitation interventions, including their type or category, dose or frequency, duration, and any modification, will be carefully recorded by blinded assessors to facilitate assessment of the comparability of co-interventions between groups.

Conventional acupuncture will be administered as part of the routine integrative treatment for stroke. The selection of acupoints for conventional acupuncture will be based on the national higher education textbook Acupuncture and Moxibustion (35) and the routine clinical practice pathway of the Department of Acupuncture, The Second Affiliated Hospital of Guangzhou University of Chinese Medicine.

Body acupuncture points are selected as follows: GV20 (Baihui), EX-HN3 (Yintang), bilateral GB20 (Fengchi), bilateral PC6 (Neiguan), bilateral GB34 (Yanglingquan), CV12 (Zhongwan), CV10 (Xiawan), CV6 (Qihai), CV4 (Guanyuan), contralateral KI17 (Shangqu), and ipsilateral ST24 (Huaroumen) and ST26 (Wailing).

Procedure: with the patient in a comfortable position, the skin at each acupoint will be routinely disinfected. Disposable sterile filiform needles (Han Yi brand, 0.25 × 25 mm, 0.25 × 40 mm, or 0.25 × 50 mm; manufacturer: Changchun Aikang Medical Devices Co., Ltd.) will be inserted perpendicularly into the selected acupoints, with needle depth adjusted according to the anatomical location of each point. After the arrival of needling sensation (deqi), a neutral manipulation (neither reinforcing nor reducing) will be applied. Needles will be retained for 20 min.

### Adjunctive Shallow Acupuncture group

Participants in the adjunctive Shallow Acupuncture group will receive the same standard medical management, nursing care, rehabilitation program, and conventional acupuncture regimen as the conventional acupuncture group. In addition, they will undergo adjunctive treatment with Shallow Acupuncture.

Selection of pathological response points: Shallow Acupuncture will target pathological response points located in the temporal and occipital regions. Consistent with the concept introduced in the Introduction, pathological response points will be operationally identified as clinically detectable reactive areas within the temporal and occipital regions. These points may present with one or more of the following features: changes in skin color (e.g., pallor or darkening), abnormal hair follicle distribution (e.g., focal areas with unusually enlarged pores), alterations in the superficial vascular network (e.g., abnormal superficial arteries or veins), changes in skin tension (e.g., increased tightness on light palpation), morphological abnormalities (e.g., palpable subcutaneous nodules or cord-like structures on deep palpation), or sensory abnormalities (e.g., tenderness, soreness, or numbness).

Definition of the Shallow Acupuncture regions: Temporal region: defined in this study as the scalp area bounded superiorly by the temporal line, inferiorly by the upper margin of the zygomatic arch, anteriorly by the frontal process of the zygomatic bone, and posteriorly by the auricle and the anterior border of the mastoid process. Occipital region: defined in this study as the scalp area bounded superiorly by a horizontal line passing through the vertex of the lambdoid suture (lambda), inferiorly by a horizontal line at the level of the spinous process of the second cervical vertebra (C2), and laterally by the mastoid process and the posterior temporal line.

Standardized identification and selection procedure: Pathological response points will be identified using a standardized procedure consisting of visual inspection, palpation, and confirmation of local sensory responses. Visual features, such as changes in skin color, will be assessed and recorded when present. However, in the temporal and occipital regions, point selection will rely primarily on palpation-based and sensory features, as defined above. A point will be considered a positive pathological response point when at least one predefined pathological response feature is present. In each session, approximately 15–20 pathological response points will be selected within the predefined temporal and occipital regions, depending on the distribution of positive pathological response points. Among positive pathological response points, those with clearly identifiable palpation-based abnormalities and/or local patient-reported sensory responses during palpation will be prioritized. The number, location, and defining features of selected points will be documented at each treatment session. All acupuncturists responsible for Shallow Acupuncture will receive standardized training in the identification and documentation of pathological response points before trial initiation.

Shallow Acupuncture procedure: Patients will be placed in a sitting position with the head slightly flexed or in the prone position. After routine skin disinfection, disposable sterile filiform needles (Han Yi brand, 0.25 × 25 mm; Changchun Aikang Medical Devices Co., Ltd.) will be inserted with a transverse or oblique Shallow Acupuncture technique at positive pathological response points within the temporal and occipital regions. The needle tip will form an angle of approximately 10°–30° with the skin surface, and the needle will be advanced subcutaneously along the direction of the lesion for about 0.2–0.6 cun, approximately 4–12 mm in this protocol. Needles will be oriented toward the presumed lesion site and retained for 20 min. During needle retention, patients will be guided to perform targeted functional exercises tailored to their neurological deficits. The key procedural parameters of Shallow Acupuncture at temporal and occipital pathological response points are detailed in Table 3.

**Table 3:** Shallow Acupuncture protocol at temporal and occipital pathological response points.

Aspect	Protocol	
Target regions	Temporal region: Superior: temporal line; inferior: superior margin of the zygomatic arch; anterior: frontal process of the zygomatic bone; posterior: auricle and anterior border of the mastoid process.	Occipital region: Superior: horizontal line passing through the vertex of the lambdoid suture (lambda); inferior: horizontal line at the level of the spinous process of the second cervical vertebra (C2); lateral: mastoid process and posterior temporal line.
Definition of pathological response points	Presence of at least one predefined feature, including abnormal skin color, abnormal pore or hair follicle distribution, superficial vascular changes, changes in skin tension, palpable subcutaneous nodules or cord-like structures, and/or local tenderness, soreness, or numbness.	
Point selection rule and dose	Approximately 15–20 pathological response points per session, depending on the distribution of positive pathological response points; among positive pathological response points, those with clearly identifiable palpation-based abnormalities and/or local patient-reported sensory responses during palpation will be prioritized.	
Needling technique	Transverse or oblique subcutaneous insertion at approximately 10°–30° toward the presumed lesion site; shallow insertion depth of about 0.2–0.6 cun, approximately 4–12 mm in this protocol; needle retention for 20 min.	
Concomitant functional training	During needle retention, patients are guided to perform task-oriented functional activities tailored to their neurological deficits, such as active shoulder and elbow movements for upper-limb weakness.	
Treatment frequency and course	One session per day, 7 days per week, for a total of 14 consecutive sessions.	

### Outcome measures

At baseline, demographic and clinical characteristics will be collected for all participants, including age, sex, educational level, occupation, and disease duration. In addition, the types and spatial distribution of pathological response points in the temporal and occipital regions will be documented.

### Primary outcome measures

The primary clinical outcome will be activities of daily living, assessed by the Barthel Index (BI).

Activities of daily living: Activities of daily living will be assessed using the BI (36), which is widely used to evaluate basic activities of daily living and functional recovery before and after treatment. The total score ranges from 0 to 100, with higher scores indicating greater functional independence and lower levels of dependence.

### Secondary outcome measures

Secondary outcome measures will include the National Institutes of Health Stroke Scale (NIHSS) and the modified Rankin Scale (mRS).

National Institutes of Health Stroke Scale (NIHSS): The NIHSS (37) is a brief, convenient, and reliable scale for assessing neurological deficits in patients with stroke. It will be used to evaluate the severity of neurological impairment and changes in neurological status over the course of the intervention.

Modified Rankin Scale (mRS): The mRS (38) is a 7-point scale (0–6) used to assess the degree of functional disability and global outcome after stroke. It primarily reflects the level of functional independence, including bodily function, mobility, and participation in activities of daily living.

### Secondary and exploratory neuroimaging outcomes

The neuroimaging outcomes will be assessed in the predefined imaging subgroup and will include indices derived from rs-fMRI and DTI analyses. rs-fMRI measures will focus primarily on resting-state functional connectivity (FC), particularly resting-state network alterations evaluated using independent component analysis (ICA). DTI measures will focus primarily on fractional anisotropy (FA), with particular attention to the corticospinal tract (CST) and other major motor-related white matter pathways. Mean diffusivity (MD) will be examined as an exploratory diffusion metric.

### Functional MRI and DTI data acquisition

All MRI data will be acquired on a 3.0 T Magnetom Prisma scanner (Siemens, Erlangen, Germany) at The Second Affiliated Hospital of Guangzhou University of Chinese Medicine (Guangzhou). Before scanning, all participants will be screened for MRI contraindications and prepared according to standard safety procedures. During image acquisition, participants will lie in the supine position, be instructed to close their eyes, relax, remain awake, and minimize head movement. Ear protection and foam padding will be used, and participants will be monitored throughout the examination to ensure safety and compliance.

A set of localizer images will first be obtained to confirm head position and slice orientation (53 slices; repetition time (TR) = 520 ms; echo time (TE) = 4.92 ms; slice thickness = 2.0 mm; field of view (FOV) = 192 × 192 mm^2^; flip angle = 60°; voxel size = 3.0 × 3.0 × 2.0 mm^3^; matrix = 256 × 256).

For structural imaging, three-dimensional T1-weighted images of the whole brain will be acquired using a gradient-echo sequence. The scan parameters will be: 192 slices; TR = 1900 ms; TE = 2.26 ms; slice thickness = 1.0 mm; FOV = 256 × 256 mm^2^; flip angle = 9°; voxel size = 1.0 × 1.0 × 1.0 mm^3^; matrix = 256 × 256.

Resting-state functional images will be collected using a T2-weighted gradient-echo echo-planar imaging (EPI) sequence for BOLD fMRI. The protocol will include 37 axial slices with TR = 2000 ms, TE = 30 ms, slice thickness = 3.0 mm, FOV = 224 × 224 mm^2^, flip angle = 90°, voxel size = 3.5 × 3.5 × 3.0 mm^3^, and a matrix of 64 × 64.

For DTI, diffusion-weighted images will be obtained with 25 slices (TR = 3,500 ms; TE = 95 ms; slice thickness = 4.0 mm; FOV = 220 × 220 mm^2^; voxel size = 1.7 × 1.7 × 4.0 mm^3^; matrix = 128 × 128).

### Patient safety

Safety will be assessed throughout the intervention period. All acupuncture procedures will be performed in strict accordance with standardized acupuncture operation guidelines to minimize the risk of adverse events. If any acupuncture-related adverse reactions occur during treatment—such as needle stagnation, vasovagal syncope (needle fainting), or subcutaneous hematoma—appropriate management will be provided immediately, the event will be graded, and detailed records will be kept. No formal data monitoring committee is planned because this is a single-center, low-risk acupuncture trial; safety will be monitored by the investigators and the ethics committee.

Adverse events will be graded as follows:

Grade 1: Safe, no obvious discomfort.
Grade 2: Mild adverse reactions, such as transient burning, itching, or pain at the acupoint after needling, which resolve spontaneously without intervention.
Grade 3: Moderate adverse reactions that require medical management, after which the patient can continue the treatment protocol.
Grade 4: Severe adverse reactions that prevent continuation of treatment and require termination of the trial for that patient.

### Clinical data analysis

All clinical statistical analyses will be performed by independent statisticians who are blinded to group allocation, using SPSS version 26.0 (IBM Corp., Armonk, NY, USA). The primary clinical analysis will focus on the between-group difference in BI change after treatment. Analyses of clinical outcomes will primarily follow the intention-to-treat principle, including all randomized participants according to their allocated group. A per-protocol analysis may also be conducted as a sensitivity analysis in participants who complete the assigned intervention. Missing data will be handled using appropriate statistical methods depending on the extent and pattern of missingness.

Continuous variables will be presented as mean ± standard deviation (SD) when normally distributed or as median with interquartile range (IQR) when non-normally distributed. Categorical variables will be summarized as frequencies and percentages. Between-group comparisons of continuous variables will be performed using the independent-samples t-test for normally distributed data or the Mann–Whitney U test otherwise. Within-group changes over time will be assessed using paired-samples t-tests or Wilcoxon signed-rank tests, as appropriate. For categorical or ordinal variables, the chi-square test, Fisher’s exact test, or rank-sum test will be used as appropriate. Clinically relevant concomitant medications and rehabilitation interventions will be summarized by group using descriptive statistics, with attention to the magnitude and clinical relevance of any between-group differences. If clinically meaningful imbalances in co-interventions are observed, these factors will be considered in the interpretation of the results, and exploratory sensitivity analyses or covariate-adjusted analyses may be performed as appropriate.

For repeated clinical assessments, changes over time and between-group differences will be further evaluated using repeated-measures analysis or an equivalent mixed-effects approach, where appropriate. The relationships between clinical outcome measures and imaging parameters will be assessed using Pearson or Spearman correlation analyses, depending on the distribution of the data. All statistical tests will be two-sided, and a *p*-value < 0.05 will be considered statistically significant.

### Neuroimaging data analysis

Neuroimaging analyses will be conducted in the predefined imaging subgroup and will be considered secondary and exploratory. Given its potential influence on rs-fMRI and DTI measures, age will be considered as a covariate in exploratory neuroimaging analyses where appropriate. Resting-state fMRI data will be preprocessed using SPM12 (Wellcome Trust Centre for Neuroimaging, London, UK) running on the MATLAB platform. The preprocessing pipeline will include slice-timing correction, head-motion correction, spatial normalization to Montreal Neurological Institute (MNI) space, and spatial smoothing.

rs-fMRI analyses will focus primarily on resting-state functional connectivity (FC). Independent component analysis (ICA) will be performed using the Group ICA of fMRI Toolbox (GIFT). The analysis will include dimensionality reduction, ICA decomposition, and component back-reconstruction. The number of independent components will be estimated according to the minimum description length criterion, and the Infomax algorithm will be used for ICA decomposition. Component stability will be evaluated using ICASSO, and components with a stability index > 0.90 will be retained. Spatial maps will then be converted into z-score maps, and functionally meaningful resting-state networks will be identified by comparison with standard network templates.

DTI data will be processed using the FMRIB Software Library (FSL, Oxford, UK). Preprocessing will include eddy-current correction, head-motion correction, and registration to standard space. DTI analyses will focus primarily on fractional anisotropy (FA), with particular attention to the corticospinal tract (CST) and other major motor-related white matter pathways. Mean diffusivity (MD) will be examined as an exploratory diffusion metric. Group comparisons of diffusion indices will be performed using the Randomise tool in FSL, with permutation-based nonparametric testing and threshold-free cluster enhancement (TFCE) for multiple-comparison correction. For imaging analyses, results will be interpreted primarily as exploratory, and corrected *p*-values < 0.05 will be considered statistically significant.

## Discussion

Brainstem infarction is clinically fulminant and often associated with poor outcomes. At present, disease-specific therapeutic strategies and high-quality clinical studies focusing on this population remain limited, highlighting the need to explore adjunctive strategies that may support functional recovery. Against this background, the present trial is designed to evaluate whether the addition of Shallow Acupuncture to routine inpatient integrative care may further improve activities of daily living and neurological recovery. In parallel, this study incorporates rs-fMRI and DTI to explore whether clinical improvement is accompanied by changes in resting-state functional connectivity and white-matter microstructural integrity.

This protocol has two main strengths. First, it focuses specifically on brainstem infarction, a clinically important but relatively under-investigated subtype of posterior circulation infarction. By focusing on this specific population, the present trial aims to provide more targeted clinical evidence in an area with substantial unmet need. Second, by jointly applying rs-fMRI and DTI, the trial provides a multimodal framework for exploring clinical–imaging associations. In this study, rs-fMRI will focus primarily on resting-state functional connectivity, whereas DTI will focus primarily on fractional anisotropy in the corticospinal tract and other major motor-related white matter pathways. Together, these modalities may help characterize functional and structural changes associated with recovery after brainstem infarction. This multimodal framework is supported by the pathophysiology of brainstem infarction and by previous evidence related to acupuncture and neural recovery.

From a vascular perspective, the brainstem is supplied mainly by the vertebral arteries, basilar artery, posterior cerebral arteries, and their branches (39). Hypoperfusion within these vessels represents a key pathological basis for the development of brainstem infarction (40). Beyond focal tissue injury, brainstem infarction may also disrupt large-scale brain network organization, thereby contributing to persistent neurological deficits (41). Accordingly, functional recovery after cerebral infarction depends not only on structural preservation but also on neuroplasticity, that is, the capacity of the brain to reorganize its function and structure through endogenous mechanisms (42). This process is closely associated with clinical recovery (43) and involves both neural remodeling in peri-infarct regions and reorganization of large-scale network connectivity (44, 45). These considerations provide a physiological rationale for investigating adjunctive interventions that may support recovery after brainstem infarction.

As described above, Shallow Acupuncture in this trial focuses on the identification and stimulation of pathological response points within the temporal and occipital regions. This lesion-directed selection strategy provides a disease-oriented therapeutic rationale for brainstem infarction. At the neurovascular level, transcranial Doppler studies have shown that acupuncture at cervical and occipital acupoints can increase mean blood flow velocity in the vertebral and basilar arteries in patients with posterior circulation ischemia (17). At the functional network level, fMRI studies have shown that acupuncture at GB20 (Fengchi) and other cervico-occipital acupoints can modulate functional connectivity among brain regions in patients with hemodynamic abnormalities of the posterior circulation (18). These findings link cervico-occipital stimulation to vertebrobasilar hemodynamics and functional network modulation. Together with our preliminary clinical observations in patients with brainstem infarction and our previous randomized clinical study of Shallow Acupuncture for posterior circulation ischemic vertigo (19), these findings provide a rationale for further evaluating Shallow Acupuncture as a disease-oriented, lesion-directed adjunctive intervention for brainstem infarction and for assessing associated changes with rs-fMRI and DTI.

rs-fMRI provides a useful tool for evaluating changes in large-scale brain network connectivity (46). In the context of brainstem infarction, previous studies have shown that abnormalities in resting-state functional connectivity are associated with functional recovery (21–23, 47). DTI, by quantifying the diffusion properties of water molecules, enables assessment of white matter tract integrity. Fractional anisotropy in the corticospinal tract has been shown to decrease after brainstem infarction, and subsequent recovery may be associated with more favorable motor outcomes (27). Accordingly, the integration of rs-fMRI and DTI in the present trial is intended to provide complementary information on brain network reorganization and white-matter microstructural integrity after brainstem infarction. By combining clinical outcomes with multimodal neuroimaging measures, this study is expected to generate preliminary evidence regarding whether adjunctive Shallow Acupuncture is associated with measurable changes at both the clinical and neuroimaging levels.

Several limitations should be acknowledged. First, this is a single-center trial with a relatively modest sample size, and the findings should therefore be interpreted as preliminary and require confirmation in larger, multicenter studies. Given the anatomical and clinical heterogeneity of brainstem infarction involving the midbrain, pons, and medulla, broad functional measures were selected to preserve comparability across the full cohort, which may have limited sensitivity to certain brainstem-specific deficits. Second, the trial adopts a pragmatic add-on design in which both groups receive standard care, routine rehabilitation, and conventional acupuncture, while Shallow Acupuncture is added only in the experimental arm. This design reflects routine inpatient practice in the acupuncture department, but it is not intended to isolate the specific effects of Shallow Acupuncture from nonspecific contextual effects; accordingly, any observed between-group differences should be interpreted primarily as evidence of adjunctive effectiveness within an integrative care framework. In addition, residual imbalance in individualized concomitant medications or rehabilitation interventions cannot be fully excluded and will be considered when interpreting the results. Third, because of the nature of the intervention, participants and acupuncturists cannot be blinded. Although outcome assessors and statisticians will remain blinded to group allocation, performance and expectation bias cannot be entirely excluded. Despite predefined operational criteria and practitioner training, residual operator-dependent judgment in identifying pathological response points cannot be fully eliminated. Neuroimaging findings should also be interpreted as exploratory.

In summary, this trial is designed to evaluate the adjunctive effectiveness of Shallow Acupuncture for brainstem infarction in routine integrative inpatient care and to explore associated changes in brain functional connectivity and white-matter microstructural integrity using rs-fMRI and DTI. The findings may provide preliminary clinical and neuroimaging evidence to inform future studies of Shallow Acupuncture in brainstem infarction and to support the development of more rigorous multicenter trials in this field.

## References

[1] Gowda SN Munakomi S De Jesus O. "Brainstem Stroke". In: StatPearls. Treasure Island, FL: StatPearls Publishing (2025)

[2] Sciacca S Lynch J Davagnanam I Barker R. Midbrain, Pons, and medulla: anatomy and syndromes. Radiographics. (2019) 39:1110–25. doi: 10.1148/rg.2019180126, 31283463

[3] Querol-Pascual MR. Clinical approach to brainstem lesions. Semin Ultrasound CT MR. (2010) 31:220–9. doi: 10.1053/j.sult.2010.03.004, 20483390

[4] GBD 2015 Mortality and Causes of Death Collaborators. Global, regional, and national life expectancy, all-cause mortality, and cause-specific mortality for 249 causes of death, 1980-2015: a systematic analysis for the global burden of disease study 2015. Lancet. (2016) 388:1459–544. doi: 10.1016/S0140-6736(16)31012-1, 27733281 PMC5388903

[5] Ng AC. Posterior circulation Ischaemic stroke. Am J Med Sci. (2022) 363:388–98. doi: 10.1016/j.amjms.2021.10.027, 35104439

[6] Huo XC Gao F. Chinese guidelines for endovascular treatment of acute ischemic stroke 2023. Chin J Stroke. (2023) 18:684–711.

[7] Sigurdsson AP Gunnarsson T Thorisson HM Olafsson IH Gunnarsson GB. Lokun í botn- og hryggslagæð heila. Sjúkratilfelli og yfirlit [occlusion of the vertebrobasilar artery. Case presentation and literature review]. Laeknabladid. (2020) 106:302–9. doi: 10.17992/lbl.2020.06.586 32491992

[8] Goyal M Menon BK van Zwam WH Dippel DW Mitchell PJ Demchuk AM . Endovascular thrombectomy after large-vessel ischaemic stroke: a meta-analysis of individual patient data from five randomised trials. Lancet. (2016) 387:1723–31. doi: 10.1016/S0140-6736(16)00163-X 26898852

[9] Winstein CJ Stein J Arena R Bates B Cherney LR Cramer SC . Guidelines for adult stroke rehabilitation and recovery: a guideline for healthcare professionals from the American Heart Association/American Stroke Association. Stroke. (2016) 47:e98–e169. doi: 10.1161/STR.0000000000000098, 27145936

[10] Zhang J Ji C Zhai X Ren S Tong H. Global trends and hotspots in research on acupuncture for stroke: a bibliometric and visualization analysis. Eur J Med Res. (2023) 28:359. doi: 10.1186/s40001-023-01253-w 37735698 PMC10512511

[11] Chinese Society of Neurology, Cerebrovascular Disease Group of the Chinese Society of Neurology. Chinese guidelines for the diagnosis and treatment of acute ischemic stroke 2023. Chin J Neurol. (2024) 57:523–59.

[12] Li T Zeng XX Lin LJ Lin WN Mao J Wang Q . Catheter balloon dilation combined with acupuncture for cricopharyngeal achalasia after brain stem infarction: a randomized controlled trial. Zhongguo Zhen Jiu. (2019) 39:1027–33. doi: 10.13703/j.0255-2930.2019.10.001, 31621251

[13] Cheng XN Lyu XH Hao PN Lang XG Liang ZJ. Clinical observation of tongue three-needle combined with Chinese herbal medicine for dysphagia in patients with pontine infarction. Shanghai J Acupunct Moxibustion. (2024) 43:129–34. doi: 10.13460/j.issn.1005-0957.2024.02.0129

[14] Cui YN. Clinical observation of crossed electro-acupuncture at cervical points for limb motor dysfunction in convalescent brainstem infarction [dissertation]. Harbin: Heilongjiang University of Chinese Medicine (2024).

[15] Baldry P. Superficial versus deep dry needling. Acupunct Med. (2002) 20:78–81. doi: 10.1136/aim.20.2-3.78, 12216605

[16] Lin J Gu Z Zhou P Huang W Ou A Zhao Q . Shallow acupuncture for chronic neck pain: a multicenter randomized controlled trial protocol with fMRI and DTI. J Pain Res. (2025) 18:1963–73. doi: 10.2147/JPR.S512989 40236722 PMC11998947

[17] Chen SW. Clinical observation on treatment of posterior circulation ischemic vertigo with Nape Seven Needles [dissertation]. Jinan: Shandong University of Traditional Chinese Medicine (2022).

[18] Yuan XJ. Multimodal MRI Study of Acupuncture at Fengchi (GB20) for Dizziness/vertigo with Posterior Circulation Hemodynamic Abnormalities [Dissertation]. Beijing: Beijing University of Chinese Medicine (2022).

[19] Liu Q. Clinical Observation on the Efficacy of Shallow Acupuncture for Posterior Circulation Ischemic vertigo [Dissertation]. Guangzhou: Guangzhou University of Chinese Medicine (2024).

[20] Woodward OB Driver I Schwarz ST Hart E Wise R. Assessment of brainstem function and haemodynamics by MRI: challenges and clinical prospects. Br J Radiol. (2023) 96:20220940. doi: 10.1259/bjr.20220940, 37721043 PMC10607409

[21] Zhang J Chang Y. Alterations of static and dynamic functional network connectivity in acute ischemic brainstem stroke. Acta Radiol. (2023) 64:1623–30. doi: 10.1177/02841851221127271, 36113019

[22] Wang Y Wang C Wei Y Miao P Liu J Wu L . Abnormal functional connectivities patterns of multidomain cognitive impairments in pontine stroke patients. Hum Brain Mapp. (2022) 43:4676–88. doi: 10.1002/hbm.25982, 35770854 PMC9491282

[23] Jiang L Geng W Chen H Zhang H Bo F Mao CN . Decreased functional connectivity within the default-mode network in acute brainstem ischemic stroke. Eur J Radiol. (2018) 105:221–6. doi: 10.1016/j.ejrad.2018.06.018, 30017284

[24] Mukherjee P Berman JI Chung SW Hess CP Henry RG. Diffusion tensor MR imaging and fiber tractography: theoretic underpinnings. AJNR Am J Neuroradiol. (2008) 29:632–41. doi: 10.3174/ajnr.A1051, 18339720 PMC7978191

[25] Li J Rong DD Shan Y Zhang M Zhao C Lu J. Brain abnormalities in pontine infarction: a longitudinal diffusion tensor imaging and functional magnetic resonance imaging study. J Stroke Cerebrovasc Dis. (2022) 31:106205. doi: 10.1016/j.jstrokecerebrovasdis.2021.106205, 34879300

[26] Huang YC Hsu TW Leong CP Hsieh HC Lin WC. Clinical effects and differences in neural function connectivity revealed by MRI in subacute hemispheric and brainstem infarction patients with dysphagia after swallowing therapy. Front Neurosci. (2018) 12:488. doi: 10.3389/fnins.2018.00488 30079009 PMC6062613

[27] Park JW Kim SH Kim YW Kim JY Park SY Son SM . Motor control via spared peri-infarct corticospinal tract in patients with pontine infarct. J Comput Assist Tomogr. (2008) 32:159–62. doi: 10.1097/RCT.0b013e31814cf231, 18303307

[28] Writing Group of the Chinese Primary Care Guidelines for Ischemic Stroke. Guidelines for primary care of ischemic stroke (2021). Chin J Gen Pract. (2021) 20:927–46.

[29] Oldfield RC. The assessment and analysis of handedness: the Edinburgh inventory. Neuropsychologia. (1971) 9:97–113. doi: 10.1016/0028-3932(71)90067-4, 5146491

[30] Zhang F Wang GL Wang LP Zhang T Wang SS. Clinical observation of He’s SanTong acupuncture method in 14 patients with Wallenberg syndrome. J Tradit Chin Med. (2017) 58:1214–7. doi: 10.13288/j.11-2166/r.2017.14.012

[31] Mayer EA Aziz Q Coen S Kern M Labus JS Lane R . Brain imaging approaches to the study of functional GI disorders: a Rome working team report. Neurogastroenterol Motil. (2009) 21:579–96. doi: 10.1111/j.1365-2982.2009.01304.x 19646070 PMC3829384

[32] Desmond JE Glover GH. Estimating sample size in functional MRI (fMRI) neuroimaging studies: statistical power analyses. J Neurosci Methods. (2002) 118:115–28. doi: 10.1016/s0165-0270(02)00121-8 12204303

[33] Szucs D Ioannidis JP. Sample size evolution in neuroimaging research: an evaluation of highly-cited studies (1990-2012) and of latest practices (2017-2018) in high-impact journals. NeuroImage. (2020) 221:117164. doi: 10.1016/j.neuroimage.2020.117164, 32679253

[34] Chinese Society of Neurology, Cerebrovascular Disease Group of the Chinese Society of Neurology. Chinese guideline for the secondary prevention of ischemic stroke and transient ischemic attack 2022. Chin J Neurol. (2022) 55:1071–110. doi: 10.3760/cma.j.cn113694-20220714-00548

[35] Wang H Du YH, editors. Acupuncture and Moxibustion. 9th ed. Beijing: China Traditional Chinese Medicine Press (2012).

[36] Mahoney FI Barthel DW. Functional evaluation: the Barthel index. Md State Med J. (1965) 14:61–5. 14258950

[37] Kwah LK Diong J. National Institutes of Health stroke scale (NIHSS). J Physiother. (2014) 60:61. doi: 10.1016/j.jphys.2013.12.012, 24856948

[38] Haggag H Hodgson C. Clinimetrics: modified Rankin scale (mRS). J Physiother. (2022) 68:281. doi: 10.1016/j.jphys.2022.05.017, 35715375

[39] Sparaco M Ciolli L Zini A. Posterior circulation ischaemic stroke-a review part I: anatomy, aetiology and clinical presentations. Neurol Sci. (2019) 40:1995–2006. doi: 10.1007/s10072-019-03977-2, 31222544

[40] Chi HY Hsu CF Chen AC Su CH Hu HH Fu WM. Extracranial and intracranial Ultrasonographic findings in posterior circulation infarction. J Ultrasound Med. (2018) 37:1605–10. doi: 10.1002/jum.14501, 29193196

[41] Hoshino H Takagi M Yamamoto Y Ishibashi Y Terayama Y Takeda H . Neurological progression and clinical outcome of branch atheromatous disease (results from the J-BAD registry). Rinsho Shinkeigaku. (2010) 50:919–20. doi: 10.5692/clinicalneurol.50.919 21921510

[42] von Bernhardi R Bernhardi LE Eugenín J. What is neural plasticity? Adv Exp Med Biol. (2017) 1015:1–15. doi: 10.1007/978-3-319-62817-2_1, 29080018

[43] Cirillo C Brihmat N Castel-Lacanal E Le Friec A Barbieux-Guillot M Raposo N . Post-stroke remodeling processes in animal models and humans. J Cereb Blood Flow Metab. (2020) 40:3–22. doi: 10.1177/0271678X19882788, 31645178 PMC6928555

[44] Han Q Xie Y Ordaz JD Huh AJ Huang N Wu W . Restoring cellular energetics promotes axonal regeneration and functional recovery after spinal cord injury. Cell Metab. (2020) 31:623–641.e8. doi: 10.1016/j.cmet.2020.02.002, 32130884 PMC7188478

[45] Olafson ER Jamison KW Sweeney EM Liu H Wang D Bruss JE . Functional connectome reorganization relates to post-stroke motor recovery and structural and functional disconnection. NeuroImage. (2021) 245:118642. doi: 10.1016/j.neuroimage.2021.118642, 34637901 PMC8805675

[46] Logothetis NK. What we can do and what we cannot do with fMRI. Nature. (2008) 453:869–78. doi: 10.1038/nature06976, 18548064

[47] Liu G Tan S Peng K Dang C Xing S Xie C . Network change in the ipsilesional cerebellum is correlated with motor recovery following unilateral pontine infarction. Eur J Neurol. (2019) 26:1266–73. doi: 10.1111/ene.13974, 31021033

